# Improving the Standard of Orthopaedic Operation Documentation Using Typed Proforma Operation Notes: A Completed Audit Loop

**DOI:** 10.7759/cureus.1084

**Published:** 2017-03-07

**Authors:** Fionn Coughlan, Prasad Ellanti, Andrew Moriarty, Nuala McAuley, Niall Hogan

**Affiliations:** 1 Department of Trauma and Orthopaedics, St James Hospital; 2 Orthopaedic Department, Cappagh National Orthopaedic Hospital

**Keywords:** audit, orthopaedic, operation, notes, typed, proforma

## Abstract

**Introduction:**

The Royal College of Surgeons (RCS) published Good Surgical Practice guidelines in 2008 and revised them in 2014. They outline the basic standard that all surgical operation notes should meet.

**Objectives:**

To retrospectively audit 57 typed orthopaedic operation notes from St. James’s Hospital in Dublin (from August to November 2015) against the RCS Good Surgical Practice guidelines published in 2014. They were then compared with the department’s previous audit of handwritten notes to complete the audit loop.

**Materials and methods:**

A total of 57 orthopaedic operation notes were audited by a single reviewer. They were prospectively collected between August and November 2015. All notes were typed on the standard St. James’s Hospital operation note proforma.

**Results:**

Of the surgeries, 89.5% were emergencies with 77.2% of them being performed by trainees. All of the operation notes were typed and signed by trainees. The procedure name, incision and closure details, tourniquet time (when relevant), and postoperative instructions were documented in 100% of the notes. In total, 80.7% had an operative diagnosis included while only 26.9% of the documentation had prosthesis serial numbers. All of the typed notes were deemed to be legible.

**Conclusion:**

The use of printed operation notes allows for improved legibility when compared to typed notes. Documentation standards remained very high in the same areas as the handwritten notes and a marked improvement was seen in areas that had been poorly documented.

## Introduction

The Royal College of Surgeons (RCS) published the Good Surgical Practice guidelines in 2008 and revised them in 2014 [1]. They outline the basic standard that all surgical operation notes should meet. Operation notes should comply as closely as possible with these guidelines, to ensure better patient care, for research, and for audit and medico-legal purposes [2]. The revised guidelines in 2014 added in a proviso that all notes should ‘preferably be typed’. Up to 20% of handwritten orthopaedic operation notes have been shown to contain illegible parts [3]. Typed notes are beneficial in that they remove the issue of illegibility. The incidence of litigation in trauma and orthopaedics is on the rise [4]. Illegible notes can have a major impact on any surgeon’s defence in medico-legal cases [5]. Typed notes afford a clear and concise operation note which can be interpreted accurately by a large number of people. Typed and handwritten proformas that are specific to specific surgeries or to surgical specialties have been shown to increase compliance with the RCS guidelines [5,6-7]. They may also assist in training surgical trainees and act as a teaching aid for procedures [8].

Several studies have audited orthopaedic operation notes to assess the quality of documentation using the British Orthopaedic Association guidelines [7,9] and using the 2008 Good Surgical practice guidelines [3,10]. This is the first study, to our knowledge, assessing the quality of documentation using the updated 2014 guidelines. We audited typed proforma-based orthopaedic operation notes, of inpatients in St. James's Hospital Dublin in 2015, in accordance with the 2014 RCS Good Surgical Practice guidelines. We then compared these notes to the handwritten notes that we audited in our previous study. This completed the audit loop for our unit [10].​

## Materials and methods

**Figure 1:**
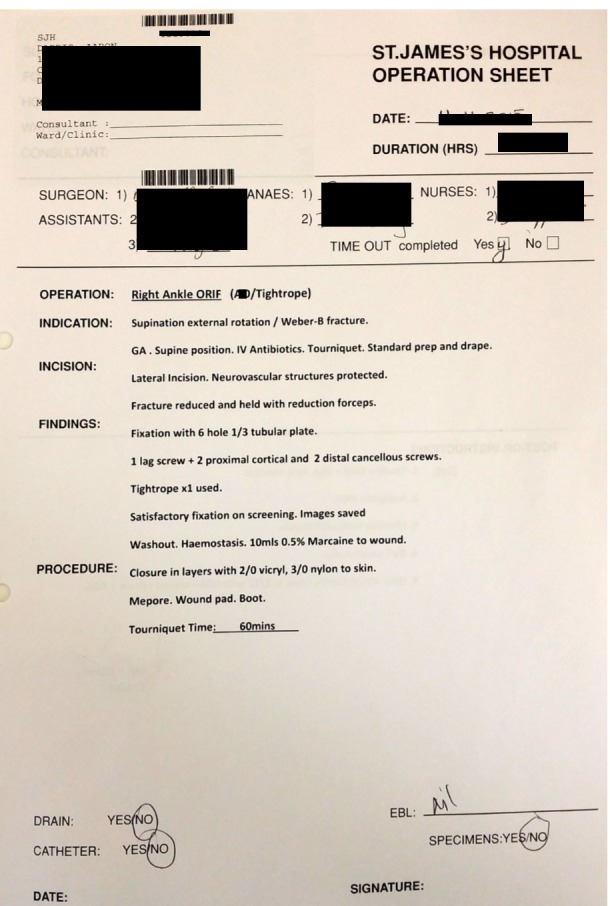
Typed Proforma Front Page

**Figure 2:**
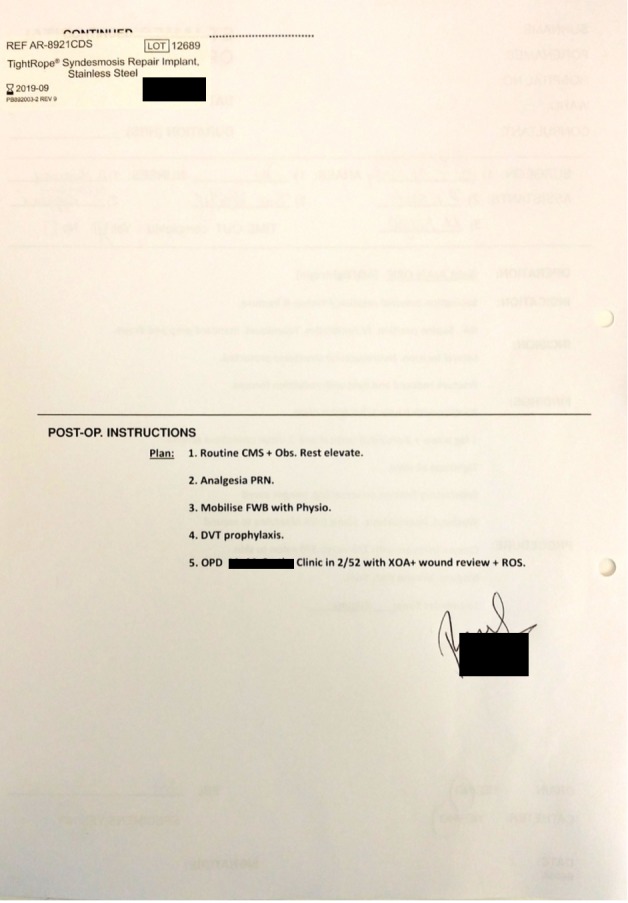
Typed Proforma Back Page

A total of 57 orthopaedic operation notes were audited by a single reviewer. They were prospectively collected between August and November 2015. All notes were typed on the standard St. James's Hospital operation note proforma. Previously, all orthopaedic notes had been handwritten on the standard surgical operation note proforma that was not orthopaedic-specific. The standard proforma contains headings for patient details, time and date, duration (hours), surgeon, assistants, anaesthetists, nurses, timeout completed (yes/no), operation, indication, incision, findings, procedure, drain (yes/no), catheter (yes/no), specimen (yes/no), and post-op instructions. The proforma is the same as used in the department’s previous audit barring the heading for tourniquet time [10]. This heading was added specifically for trial use in orthopaedic surgeries and in these typed notes. It is not present on the proforma for other surgical specialties and is only added when the note is being typed (Figures 1-2).

The operation notes were then audited against the headings used in the 2014 Good Surgical Practice guidelines: date and time of surgery, the surgeon, the procedure, elective or emergency indication, operative diagnosis, incision details, signature, closure details, tourniquet time, post-op instructions, complications, prostheses, and serial numbers [1]. The operation notes were then compared with the handwritten notes from our previous study to assess whether the standard of the documentation had improved our unit (Table 1). 

**Table 1:** Operations Performed

SITE	PROCEDURES	ELECTIVE	EMERGENCY	TOTAL
METACARPAL/DIGITS	MUA and K-wires	0	5	5
ANKLE	ORIF*/Arthroplasty	0	7	7
HIP	Hemiarthroplasty/THR*/IM nail*/DHS*/removal of metal	3	16	19
OLECRANON	ORIF	0	3	3
RADIUS	ORIF	0	10	10
HUMERUS	ORIF	0	4	4
KNEE	Washout/arthroscopy	2	0	2
TIBIA	ORIF/IM nail	0	2	2
CLAVICLE	ORIF	0	2	2
PATELLA	Tension band wiring	0	1	1
ACHILLES	Repair	1	0	1
ULNA	ORIF	0	1	1
TOTAL		6	51	57

Two independent medical doctors (non-surgeons) and one theatre nurse then reviewed the typed notes and hand-written notes, to evaluate their legibility. They reviewed the notes based on the legibility of the date, postoperative instructions, diagnosis, prosthesis used, and the procedure name itself. If any of these were illegible, then the note was deemed to be illegible.

## Results

All 57 notes were typed on the St. James's Hospital operation sheet (Figures 1-2). Emergency procedures constituted a majority 89.5% (n=51) with only 10.5% (n=6) of the surgeries being elective in nature.

The date was documented in 100% of the operation notes while only 63.2% contained the operation time/duration (n=36). Consultants performed 22.8% of the surgeries (n=13) with the remaining 77.2% being performed by trainees (n=44). All of the operation notes were typed and were signed by the trainees (n=57).

The procedure name, incision and closure details, tourniquet time (when relevant), and postoperative instructions were documented in 100% of the notes (n=57). Postoperative instructions were documented in the correct place 30.5% of the time. Only 80.7% had the operative diagnosis documented (n=46), but, where it was documented, it was documented in the correct place 100% of the time. While 98.2% of the operation notes had documented the type of prosthesis used (n=52) only 26.9% had the serial numbers attached to the operation notes (n=14). Of the handwritten notes, from the previous audit, the first 57 notes were selected and reviewed by two independent non-orthopaedic doctors and one theatre nurse. Of these handwritten notes, 14% were deemed illegible by the reviewers (n=8) while 100% of the typed notes were found to be legible. These results were then compared with the results from our previous audit, as shown in Table 2. 

**Table 2:** Comparing Handwritten and Typed Notes

Heading	Handwritten % documented	Typed % documented
Date	100	100
Duration	1.6	63.2
Elective/Emergency	0	100
Surgeon and assistants	100	100
Procedure name	100	100
Signature	100	100
Complication	100	100
Operative diagnosis	74.6	80.7
Incision	81.7	100
Tourniquet time	0	100
Closure	98.3	100
Post op orders	96.3	100
Prosthesis named	79.6	98.2
Serial numbers	30	26.9

## Discussion

Orthopaedic operation notes in St. James's Hospital are typed or written on the standard surgical proforma used by all surgical specialties. The specific headings added into the orthopaedic typed notes allowed for better documentation in the operation notes. These findings correlate well with the findings of Abbas, et al. (2016) who used specific proformas for laparoscopic appendicectomies to show a significant improvement in operation note compliance with the RCS guidelines [8]. They also showed an improvement in overall legibility when using procedure specific proformas.

Orthopaedic-specific headings in these typed notes, such as tourniquet time, improved documentation in this area when compared with the handwritten version on the basic proforma. The orthopaedic proforma used in Sheffield previously highlighted how the addition of these specialty-specific headings improved documentation [7]. Proforma benefits pertaining to operative documentation have been highlighted in other surgical specialties previously [11-12]. Shah, et al. have shown statistically significant improvement in orthopaedic operation note documentation when using the British Orthopaedic Association guidelines and the RCS guidelines as a template for their total knee replacement (TKR) and total hip replacement (THR) operation notes [6].

Singh, et al. have shown that the quality of operation note documentation can be improved by simply highlighting the deficient areas in the previous notes [13]. This can be seen in the improved documentation of tourniquet time in this audit compared to the previous audit in our unit (Table 2) [10].

A study by Sweed, et al., in their orthopaedic department, demonstrated similar deficient areas in their operation note documentation, particularly the documentation of tourniquet time [3]. The addition of a heading for tourniquet time in the typed notes has shown a noticeable improvement in documenting this particular area. Our previous audit had highlighted the poor documentation of tourniquet time in orthopaedic notes in St. James's Hospital [10]. The improved documentation of tourniquet time may be a direct result of the typed notes with a specific heading. But it is likely to be a combination of this along with the feedback received from the previous audit.

When comparing the two sets of notes, our unit performed very well in documenting the date, surgeon, procedure name, and complications. They were documented in 100% of the handwritten notes and the typed ones. Every single note audited was signed by the author. The typed notes performed better in most other areas, with 100% containing correct documentation of the incision used, closure, tourniquet time, and postoperative orders. Each typed note stated whether the procedure was elective or an emergency, which was not highlighted in any of the handwritten notes. Operative diagnosis was documented in 80.7% of the typed notes, compared to just 74.6% of the handwritten ones. The prosthesis used was named in 98.2% of the typed notes; this was present in only 79.6% of the handwritten notes. The duration of the procedure was typed on 63.2% of the typed notes, it was only present in 1.6% of the handwritten notes.

All of the typed notes were legible; this corresponds with the results presented by Ghani, et al. [14]. They found a major difference in the legibility between handwritten (66% legible) versus typed notes (100% legible) [14]. Of the notes reviewed for this paper, 86% of the handwritten notes from our previous audit were deemed legible by our three independent reviewers.

Serial numbers and the type of prostheses used remains an area where documentation can be improved upon greatly. Although the typed notes contained a better standard of naming the prosthesis used (98.2% vs. 79.6%), the serial numbers and/or implant labels were rarely attached or documented. Only 26.9% had serial numbers documented compared to 30% in the previous study. A heading or specific textbox for the serial numbers may present a solution to this and one that would be easy to implement. Shah, et al. also reported low documentation numbers for serial numbers of prostheses used in orthopaedic surgeries [6]. Their numbers were lower initially but did improve on re-audit whereas our numbers remained low.

Electronic operation notes present a future possibility for further auditing and reviews. They present many advantages over handwritten and typed notes. Electronic notes can be accessed remotely, updated at different intervals, can hold templates for common procedures, and be accessed by multiple people at any given time. Typed and handwritten paper notes are susceptible to being misplaced or lost with no method of reproducing the information they contain. The issue of serial numbers from implants remains as these are normally only attached to the original operation note document.

While electronic operation notes are becoming increasingly more popular and the standard in many centres, many more centres without the same resources and facilities continue to rely on handwritten typed notes. The use of the proforma and specific headings is shown here to be beneficial in improving the standard of operation notes and can be implemented by centres that do not have access to electronic notes. Proformas can act as aide-memoires for surgeons and this alone has been shown to improve documentation [13]. Barritt, et al. compared both handwritten and electronic notes in their study and found that computerised proformas should be used where possible. They do state that for electronic and typed notes to be used, the surgeon should have easy access to a computer workstation and a means of producing a hardcopy [15].

Studies have shown the usefulness of a specific computer software programme to standardise operation notes in an electronic format and to keep a database. Dukic, et al. demonstrated this in urology using their specific software [16]. The issue with purchasing and deploying such software could be limited by cost and hardware in some units. The existing computers may not be compatible with the software programme and a hospital’s own existing health applications may not communicate effectively with it. Such IT infrastructure investment may be beyond the scope of some units.

Limitations of this study include the small numbers of operation notes available to audit. The typed proformas were not the standard for all notes written in our orthopaedic department during the study time; therefore, the numbers of notes gathered for auditing purposes were limited to those that were typed. Allowing for these limitations, the data presented highlights the areas of improvement and ongoing weakness' in the St. James's Hospital orthopaedic department regarding the documentation of the operation notes. It represents the closure of the audit loop, with many areas seeing improved documentation, while highlighting the areas that remain poorly recorded.

## Conclusions

The use of printed operation notes allows for improved legibility when compared to typed notes. Documentation standards remained very high in the same areas as the handwritten notes, and a marked improvement was seen in most areas that had been poorly documented. The greatest improvement was seen in documenting tourniquet time and the specific heading on the proforma certainly contributed to this. Operation duration documentation, while still in need of improvement, was much better when compared to the handwritten notes.

Serial numbers of the prostheses used remain an area of inadequate documentation, one that should be addressed. A specific heading for their inclusion may prove useful. Such was the case for tourniquet time and the improvement in documentation in this area is evident.

The typed notes showed a much higher compliance with the RCS Good Surgical Practice guidelines. These results all favour the introduction of typed notes in place of handwritten ones in our orthopaedic department. 
